# Effect of Temperature and Hold Time of Induction Brazing on Microstructure and Shear Strength of Martensitic Stainless Steel Joints

**DOI:** 10.3390/ma11091586

**Published:** 2018-09-01

**Authors:** Yunxia Chen, Haichao Cui

**Affiliations:** 1 School of Materials Science and Engineering, Shanghai Dianji University, Shanghai 201306, China; cyx1978@yeah.net; 2 Shanghai Key Laboratory of Materials Laser Processing and Modification, Shanghai Jiao Tong University, Shanghai 200240, China

**Keywords:** induction brazing, elements diffusion, microstructural evolution, shear strength, stainless steel

## Abstract

1Cr12Mo martensitic stainless steel is widely used for intermediate and low-pressure steam turbine blades in fossil-fuel power plants. A nickel-based filler metal (SFA-5.8 BNi-2) was used to braze 1Cr12Mo in an Ar atmosphere. The influence of brazing temperature and hold time on the joints was studied. Microstructure of the joints brazed, element distribution and shear stress were evaluated at different brazing temperatures, ranging from 1050 °C to 1120 °C, with holding times of 10 s, 30 s, 50 s and 90 s. The results show that brazing joints mainly consist of the matrix of the braze alloy, the precipitation, and the diffusion affected zone. The filler metal elements diffusion is more active with increased brazing temperature and prolonged hold time. The shear strength of the brazed joints is greater than 250 MPa when the brazing temperature is 1080 °C and the hold time is 30 s.

## 1. Introduction

1Cr12Mo stainless steel is a modified material made by appropriately increasing the content of Mo to hold the temper brittleness on the base of AISI 403. As a martensitic heat-resistant stainless steel with good creep strength and moderate corrosion resistance, 1Cr12Mo stainless steel is widely used for intermediate and low-pressure steam turbine blades in fossil-fuel power plants. Its mechanical properties, fatigue resistance, and corrosion resistance have been researched [1,2,3,4]. However, most of these studies are limited to traditional processing technology, such as furnace and vacuum brazing [5]. Compared with vacuum brazing, induction brazing is a faster and more effective technique, which provides a fast and controllable method of heating to help elements dissolution, diffusion, and chemical reaction between the base metal and the filler metal. The heating rate of induction brazing can reach 100 °C/s, which is important to avoid liquation of the braze alloy with different solidus and liquidus temperatures [6].

For excellent performance of the brazed joint, nickel-based filler metal is often used in high-temperature alloy induction brazing. B, Si, and other elements are added to the filler metal to lower its melting point temperature and improve its liquid flow rate. However, B and Si in the filler metal can react with some metallic elements and form high hardness brittle intermetallic compounds, usually located in the diffusion affected zone of the welded joints. These intermetallic compounds have adverse effects on joint performance. The brazing temperature and hold time at high temperature have a critical influence on the diffusion of B and Si. Proper brazing temperature and hold time are helpful to the diffusion of B and Si, and the diffusion between the filler metal and the base metal [7]. Compared with BMn50NiCuCrCo and BNi82CrSiBFe filler metals, SFA-5.8 BNi-2 filler metal is the best for stainless steel brazing because of its distinct weldability [8]. In this study, induction brazing of 1Cr12Mo using a nickel-based brazing alloy, BNi-2, was investigated. Both the microstructural evolution and shear strength of the brazed joint are evaluated.

## 2. Experimental Procedure

**Figure 1:**
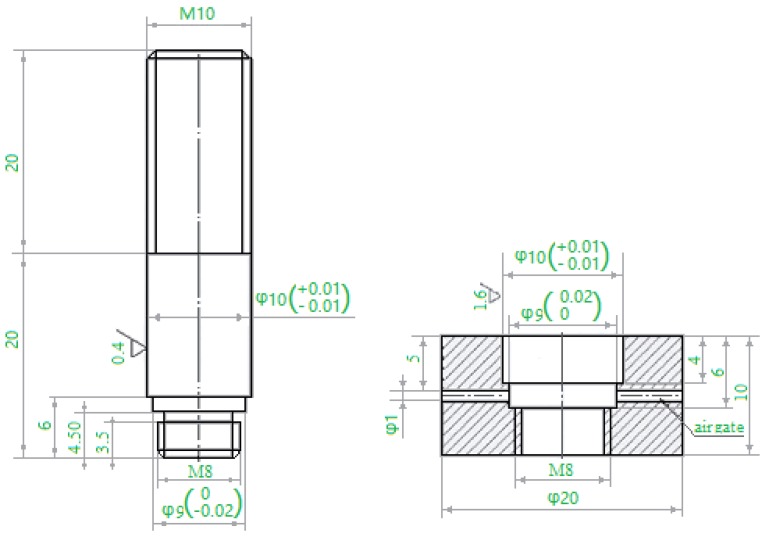
The brazed specimen for shear strength test (units: mm).

**Figure 2:**
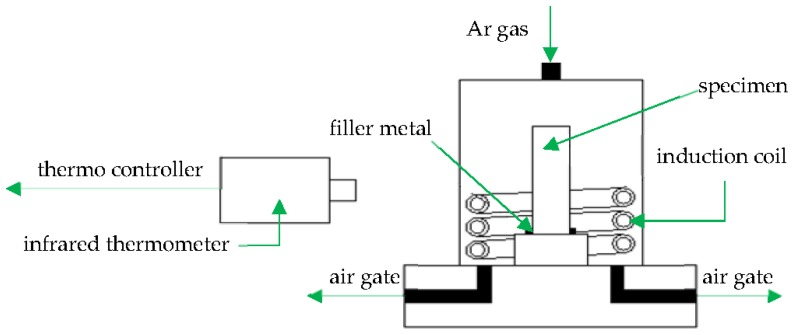
Schematic illustration of the induction brazing.

1Cr12Mo stainless steel was used as the base metal, with the chemical composition in weight percent of Cr (11.50~13.00), Ni (0.30~0.60), Mo (0.30~0.60), Mn (0.30~0.50), C (0.10~0.15), Si (0.05), P (0.035), S (0.030), and Fe (balance) according to the national (Chinese) standard GB8732. A nickel-based filler metal, BNi-2, containing in weight percent Cr (6.0~8.0), B (2.75~3.5), Si (4.0~5.0), Fe (2.5~3.5), Ni (balance) was chosen. The filler metal was in powder form with the granule size about 400 mesh. The base metal was processed to shear specimens described in the national (Chinese) standard GB11363-89, then cleaned using an ultrasonic bath and acetone solvent, and dried with hot air. Before brazing, the shear specimens were assembled as shown in Figure 1. The gap between brazed materials was 2 μm and sufficient filler metal was put on the packing place. To prevent the powder from running away, some alcohol was used during brazing. Figure 2 shows a schematic illustration of the induction brazing. A HX-GP-25 type high-frequency inductor was used as the heating equipment, and the heating current was 600 A. A high speed infrared temperature measuring instrument (Kleiber KMGA740, Kleiber, Allgäucity, German) was used to measure and record the brazing temperature. Due to the low content of Cr elements in BNi-2, to prevent oxidation the induction brazing was performed in an Ar atmosphere, and the Ar gas flow rate was 25 L/min. The brazing temperatures were 1050, 1080, 1120 and 1150 °C. The holding times were 10 s, 30 s, 50 s, and 90 s respectively.

The brazed samples were cut using a metallographic sample cutting machine, then executed in accordance with the standard metallographic procedure. The cross section of the brazed joint was examined using the JSM-7600 UHR thermal field emission scanning electron microscope (JEOL, Tokyo, Japan) with an operating voltage of 15 kV. To evaluate the bonding strength of the base metal and the filler metal, the shear test was conducted. The shear test piece was drawn by a universal testing machine (Zwick, Ulm-Einsingen, German) with a constant speed of 1 mm/min at room temperature.

## 3. Results and Discussion

**Figure 3:**
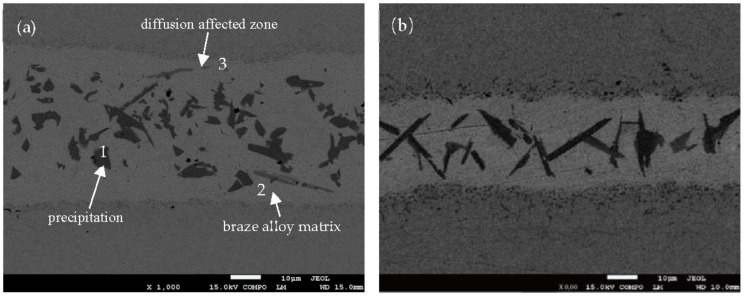

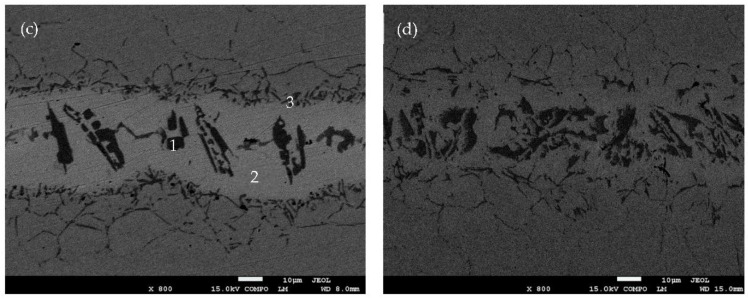
The SEM backscattered image of BNi-2 brazed at 1120 °C for (**a**) 10 s, (**b**) 30 s, (**c**) 50 s, (**d**) 90 s.

Figure 3 shows the SEM backscattered image of BNi-2 brazed at 1120 °C for 10 s, 30 s, 50 s, and 90 s respectively. The distribution of the elements in the joint can be observed in the backscattered image. The specimen areas containing high-atomic number elements appear light, while the areas containing low-atomic number elements appear dark. Based on this information, it is clear that the elements distribution of the joint is not uniform. Due to the rapid heating and cooling rate in the induction brazing, there is not enough time for the elements to distribute to equilibrium. As a result, different phases generated in the joint.

As shown in Figure 3, the bond region consists of three parts: the matrix of the braze alloy, the precipitation, and the diffusion affected zone. It has been reported that the matrix of the braze alloy is the isothermally solidified zone (ISZ) formed by isothermal solidification during the holding time [9,10]. The microstructure of the ISZ is γ solid solution (which solutes the rich Ni) and free γ’ precipitates. The precipitation in the middle of the joint is the athermally solidified zone which formed at the end of the solidification and is controlled by added elements to depress the melting point [11]. The diffusion affected zone consists of CrB, due to B diffusion and strong metal compounds for Cr and B.

Figure 3 also proves that the diffusion affected zone is more active as holding time increases. When the holding time is only 10 s, as shown in Figure 3a, there is almost no reaction layer between the base metal and the filler metal. When the holding time is prolonged to 30 s, little reaction layer can be observed (see Figure 3b). When the holding times are 50 s and 90 s, a net structure (see Figure 3c,d) formed in the diffusion affected zone, which has been reported as enhancing the joining strength of the base metal and the filler metal [12]. At the same time, the area of athermally solidified zone decreased.

**Figure 4:**
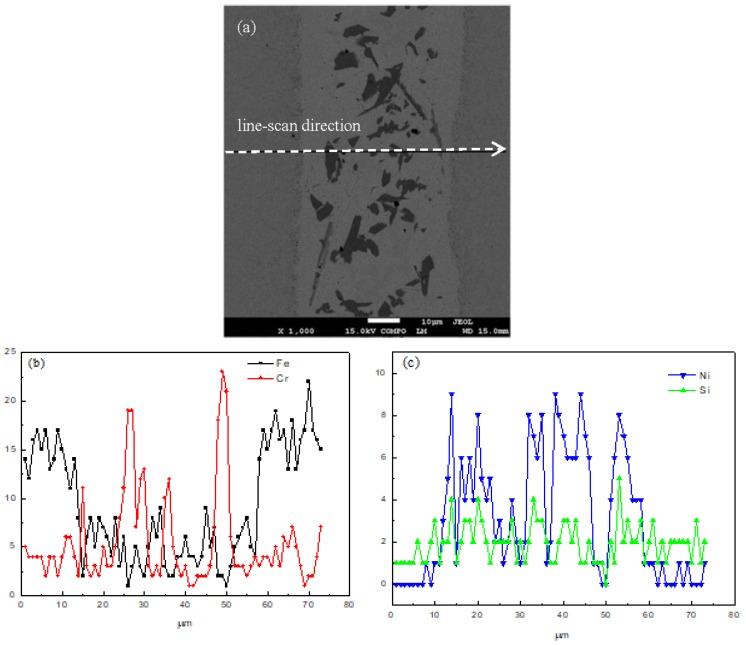
Line-scan analysis of the joint brazed at 1120 °C for 10 s, (**a**) line-scan direction, (**b**) content of Fe and Cr, (**c**) content of Ni and Si.

Figure 4 shows the elements distribution of the joint using line-scan analysis of the brazing temperature at 1120 °C for 10 s. From the base metal to the filler metal, the content of Fe and Cr decrease while the content of Ni and Si increase. The reason is that there is an interdiffusion between the base metal and the filler metal. When the scanning line reaches the precipitation, the content of Fe and Ni decrease sharply, while the content of Cr increases to maximum. Based on the principles of SEM backscattered images, it can be determined that there is B element in the precipitation. Therefore, the precipitation is identified as CrB.

Figure 3a,c show the chemical analysis of different phases in the joint brazed at 1120 °C for 10 s and 50 s respectively. There are three different phases observed in the joint. Their corresponding chemical compositions are shown in Table 1 and Table 2. It suggests that the high brazing temperature will result in some Fe atoms melting from the base metal to the joint, so there are a few Fe atoms detected in the joint. Meanwhile, the different chemical compositions of the phases in the two joints result from the different holding times during the brazing procedure. When the brazing temperature is at 1120 °C for 10 s, the precipitation in the joint (point 1 in Figure 3a) is CrB without other elements. This result is consistent with the line-scan result mentioned above. The probable reason is that the precipitation was formed in the althermal solidification at the end of the isothermal solidification. According to the phase diagram, the solubility of B in Ni decreased at the end of the isothermal solidification, then the B element was left in the liquid and resulted in the formation of CrB. With the prolonging of the holding time, more Fe atoms dissolved into the joint. The precipitation at point 1 in Figure 3c, consists of B, Cr, Fe and Ni (as shown in Table 2), which is different from the precipitation at point 1 in Figure 3a. The differences in the matrix of the joints are the content of Fe, Si and B. When the holding time is prolonged, more Fe melts into the joint, and more Si and B atoms diffuse. Due to the atom size of B being smaller than that of Si, the diffusion rate of B is faster than that of Si, and there is no B element detected at point 2 in Figure 3c. Point 3 in Figure 3a is the diffusion affected zone between the base metal and the filler metal. The composition reveals the diffusion of Ni and Si atoms. Point 3 in Figure 3c is a precipitation that primarily comprises B, Cr and Fe. As reported, B diffuses into the base metal to form the intermetallics along the grain boundaries of the base metal [13].

**Figure 5:**
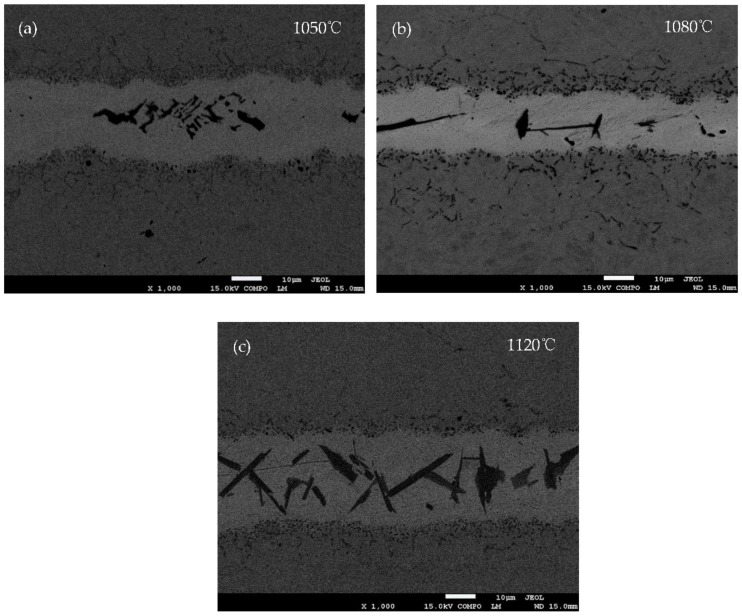
The SEM backscattered images of BNi-2 brazed at (**a**) 1050 °C, (**b**) 1080 °C and (**c**) 1120 °C for 30 s.

Figure 5 shows the SEM backscattered images of the joints brazed at 1050, 1080 and 1120 °C for 30 s, respectively. The diffusion affected zone (see Figure 5a) is not obvious, but is quite clear in Figure 5b,c. The shape of the boride phase also varies with the brazing temperature. The higher the brazing temperature, the easier the boride phases achieve phase equilibrium.

**Figure 6:**
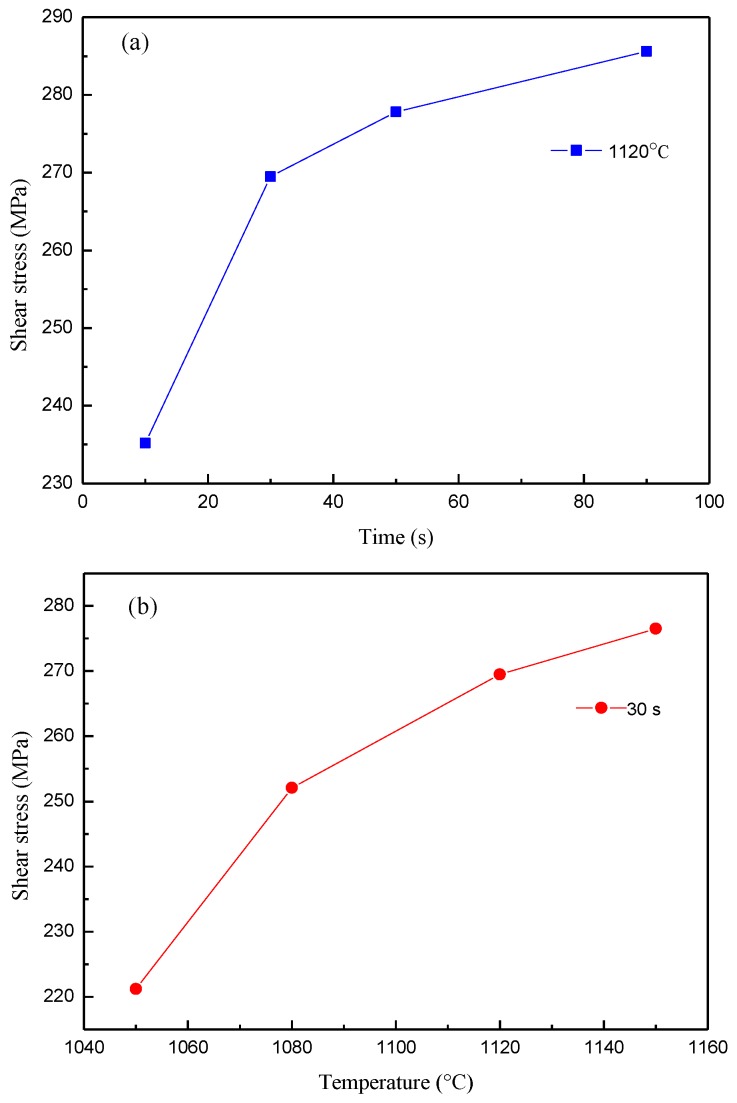
The shear test results of the brazed joints, (**a**) at 1120 °C, (**b**) for 30 s.

Table 3 shows the shear test results of brazed joints for varying brazing parameters. Most shear stress values of brazed joints are above 250 MPa, except for test samples 1 and 3; the former was brazed at a low temperature (1050 °C) and the latter was brazed for a short time (10 s). Figure 6a,b show the variation of shear stress for different brazing parameters. In Figure 6a, when the brazing temperature is 1120 °C, the shear stress of the brazing joint increases with the prolonging of the holding time. However, the increase of shear stress is not obvious when the holding time exceeds 50 s. The shear stress of the brazing joint increases with the increasing brazing temperature when the hold time is 30 s, as shown in Figure 6b. The microstructure of the joint is an indicator for its mechanical properties. The different holding time and brazing temperature that resulted in varying boride phases in the joint, which have a slight effect on the shear stress in the joint. However, the shear stress is heavily dependent on the shape of the diffusion affected zone and whether the chemical composition content in the matrix of the braze joint can easily achieve phase equilibrium [14].

## 4. Conclusions

The induction brazing of 1Cr12Mo martensitic stainless steel with nickel-based filler metal (SFA-5.8 BNi-2) in an Ar atmosphere was performed in this paper. The effect of temperature and hold time of induction brazing on microstructure and shear strength has been discussed. The research results show that the brazed joint consists of three parts, the matrix of the braze alloy, the precipitation and the diffusion affected zone. With increases of temperature and holding time, the strength of the brazed joint was increased. The shear strength of the joints brazed is 285.6 MPa when the Ar gas flow rate is 25 L/min, the heating current is 600 A, the brazing temperature is 1120 °C, and the holding time is 90 s.

## Figures and Tables

**Table 1:** The chemical compositions labeled 1,2,3 in Figure 3a.

Element	1		2		3	
	Wt %	At %	Wt %	At %	Wt %	At %
Ni	0.33	0.11	56.38	49.09	11.08	10.45
Cr	58.07	22.54	2.84	2.79	9.63	10.25
Fe	-	-	34.33	31.42	78.57	77.88
Si	0.29	0.21	4.75	8.65	0.72	1.42
B	41.31	77.13	1.7	8.05	-	-

**Table 2:** The chemical compositions labeled 1,2,3 in Figure 3c.

Element	1		2		3	
	Wt %	At %	Wt %	At %	Wt %	At %
Ni	3.46	1.05	48.09	45.43	1.74	0.47
Cr	24.32	8.31	4.08	4.36	5.17	1.57
Fe	21.13	6.72	45.06	44.75	31.95	9.01
Si	-	-	2.77	5.46	0.16	0.09
B	51.1	83.93	-	-	60.98	88.86

**Table 3:** Shear test results of the brazed joints for different process parameters.

No.	Temperature (°C)	Time (s)	Shear Stress (MPa)
1	1050	30	221.2
2	1080	30	252.1
3	1120	10	235.2
4	1120	30	269.5
5	1120	50	277.8
6	1120	90	285.6
7	1150	30	276.5
